# Establishment of a mouse embryo bank at ICTB/FIOCRUZ: vitrification of genetically modified strains

**DOI:** 10.1590/1984-3143-AR2025-0013

**Published:** 2025-11-10

**Authors:** Isabella de Moura Folhadella Pires, Janaína Barcelos Porto Ferreira, Luciene Paschoal Braga Dias, Cristiano Magalhães Ferreira, Alessandra de Almeida Ramos, Paulo César da Silva Souza, Thaís Malheiros Torres, Fabiana Batalha Knackfuss

**Affiliations:** 1 Instituto de Ciência e Tecnologia em Biomodelos, Fundação Oswaldo Cruz, Rio de Janeiro, RJ, Brasil; 2 Faculdade de Veterinária, Universidade do Grande Rio, Duque de Caxias, RJ, Brasil

**Keywords:** mice, vitrification, transgenic, embryos

## Abstract

To establish a mouse embryo bank at the Institute of Science and Technology in Biomodels, Oswaldo Cruz Foundation (ICTB/FIOCRUZ), embryos from genetically modified strains were vitrified. The strains included B6.129SVEV-CCBP2 (D6), B6.129P2-Nos2 (Nos2), B6.129S2-Cd28 (Cd28), B6.129P2-Ccl3 (Ccl3), B6.129S2-Alox5 (Alox5), B6.129P2-Ccr2 (Ccr2), B6.129P2-Ccr5 (Ccr5) and B6.129S1-Tlr6 (Tlr6). To accomplish this, the animals were superovulated and mated, and their embryos were collected and vitrified. The success of the technique was evaluated by examining the development of the embryos through thawing and in vitro culture, comparing them to a control group. The results were analyzed using percentages, Tukey's t-test, and Analysis of Variance. The embryonic development percentages for the different strains were as follows: D6 (55%), Nos2 (24.7%), Cd28 (45.8%), Ccl3 (50%), Alox5 (4.8%), Ccr2 (66.7%), Ccr5 (63.04%) and Tlr6 (52.8%). Significant differences were observed between the strains Nos2 (p=0.0434), Cd28 (p=0.034), Ccl3 (p=0.0006), and Alox5 (p=0.0166) compared to their respective control groups. In conclusion, the strains Ccr2 (p= p=0.0889), Ccr5 (p=0.0806), D6(p=0,0685) and Tlr6 (p=0.0806) demonstrated favorable results in terms of the vitrification protocol and subsequent embryonic development, as they did not significantly differ from the control groups.

## Introduction

Institute of Science and Technology in Biomodels (ICTB), a technical-scientific unit of the Oswaldo Cruz Foundation, plays a crucial role in meeting the demand for laboratory animals in Brazil. Rodents, particularly mice (*Mus musculus*), are the most widely used animal models in biomedical research. The advent of molecular genetics technologies has facilitated the development of genetically modified mouse models, which have greatly contributed to both basic and applied studies (Onos et al., 2016). Currently, there are over 35.000 mouse strains available for research purposes. However, maintaining the characteristics of these strains is a significant challenge within animal breeding and research facilities due to the potential occurrence of spontaneous mutations in each generation. Moreover, the risk of accidents such as activist invasions, fires, and genetic contamination further highlights the importance of safeguarding these valuable genetic resources.

In this context, germplasm banks assume a strategic role in animal facilities, particularly, as they enable the preservation of genetic heritage while maintaining optimal genetic and sanitary standards. Establishing germplasm banks offers several advantages, including reduced costs associated with the creation and maintenance of a large number of breeding strains. It also facilitates the efficient supply of animals to researchers, both domestically and internationally. The formation of germplasm banks typically relies on cryopreservation techniques (Whittingham et al., 1972; Wilmut, 1972), which involve lowering the temperature to halt metabolic reactions in cells, tissues, or organs, thereby allowing long-term storage (Mullen and Critser, 2007; Amorim et al., 2011a).

Consequently, the use of suitable cryopreservation protocols tailored to specific cell types or tissues is crucial for the successful implementation of germplasm banks in animal facilities (Byers et al., 2006; Dinnyés et al., 1995). However, temperature fluctuations during cooling and rewarming can result in cryoinjury and adversely affect viability (Mullen and Critser, 2007; Amorim et al., 2011b). Embryos are the most suitable structures for establishing germplasm banks due to their diploid state. Nevertheless, it has been observed that different strains respond differently to cryopreservation methods, indicating that genotype significantly influences the in vitro survival of frozen embryos (Dinnyés et al., 1995; Byers et al., 2006).

Variations in the results of in vitro culture of cryopreserved embryos have also been observed in genetically modified mouse strains at ICTB (personal communication). One of the primary challenges is the collection of a sufficient number of embryos for effective preservation, as some strains have low rates of viable embryos (Glenister and Thornton, 2000) or are influenced by genetic background (Takahashi and Liu, 2010). In a study conducted by Dinnyés et al.(1995), the authors evaluated the effects of the genotypes of four mouse strains (C57Bl/6N, BALB/Cann, DBA/2N, and C3H/HeN) subjected to two different embryo cryopreservation methods. The results indicated that genotype significantly influenced the in vitro survival of embryos, with vitrification demonstrating better pregnancy rates compared to slow freezing.

Despite the challenges involved, cryopreservation is an invaluable tool for preserving murine genetic resources, disseminating mouse models through frozen embryos, and ensuring the sanitary quality of animals (The Jackson Laboratory, 2018). The objective of this study was to evaluate the viability and embryonic development of vitrified embryos using the Tsang and Chow (2009) technique as a means to initiate the establishment of the genetically modified mouse embryo bank at ICTB. Additionally, we aimed to compare the in vitro embryonic development of different genetically modified mouse strains that demonstrated better results in vitrification.

## Methods

### Strains of cryopreserved embryos

The genetically modified strains embryos used and respective control for the study were B6.129SVEV-CCBP2(D6) (n=31/74), B6.129P2-Nos2 (n=78/155), B6.129S2-Cd28 (n=95/79), B6.129P2-Ccl3 (n=215/95), B6.129S2-Alox5 (n=129/152), B6.129P2-Ccr2 (n=75/37), B6.129P2-Ccr5 (n=75/37) and B6.129S1-Tlr6 (n=55/85). These strains were present at the Institute of Science and Technology in Biomodels (ICTB).

### Superovulation of female embryo donors

Female mice aged 3 to 7 weeks were hormonally stimulated with 5.0 IU of eCG/IP (Novormon®, Schering-Plough) followed by 5.0-7.5 IU of hCG/IP (Chorulon®, Intervet) 46 hours later. The females were then mated with proven fertile males. The presence of a vaginal plug was checked the next morning to confirm pregnancy.

### Embryo collection by washing the uterine tube

After 24 hours of confirming the presence of a vaginal plug, the pregnant females were euthanized by cervical dislocation. Their fallopian tubes were collected and placed in petri dishes (60x15 mm). The infundibulum region of the fallopian tubes was located under a stereoscopic microscope (200X), and M2 medium (Sigma-Aldrich) was used to wash the region. The collected embryonic structures were placed in drops of 50µL of M2 medium for further classification.

### Vitrification of embryos

The embryos classified as viable 2-cell embryos were subjected to vitrification using the Tsang technique. The process involved the placement of a drop of M2 medium (20µL), a drop of pre-vitrification solution (composed of ethylene glycol 10 µL 10%, DMSO 10 µL 10%, and M2 80 µL 80%), and a drop of vitrification solution (composed of ethylene glycol 15 µL 15%, DMSO 15 µL 15%, FS (Ficoll PM70 0.3 g + Sucrose 0.17 g in PBS pH 7.4, filtered) 60 µL 60%, and M2 10 µL 10%) in a petri dish. The embryos were transferred sequentially between the drops at 30-second intervals. Finally, the embryos were placed on vitrification spatulas and immediately submerged in liquid nitrogen for 5 seconds. The spatulas containing the embryos were then stored in 0.5mL straws, which were cooled and stored in a liquid nitrogen tank at -196 °C.

### Embryo thawing

For embryo thawing, a 200µL drop of 0.5M sucrose (in M2), a 200µL drop of 0.25M sucrose (in M2), and a drop of M2 were placed in a petri dish. The cryopreserved embryos were removed from the liquid nitrogen tank and exposed to room temperature for 5 seconds. Under a stereoscopic microscope, the embryos were transferred to the 0.5M sucrose drop for 2 minutes, then to the 0.25M sucrose drop for another 2 minutes. Subsequently, the embryos were washed twice in M2 medium and once in M16 medium to prepare them for in vitro culture.

### *In vitro* culture

The embryos were cultured in drops of 50 µL of M16 medium supplemented with bovine serum albumin (BSA) free of fatty acids. The culture was carried out in an incubator at 37 °C with 5% CO_2_. After this period, the embryos were classified based on their development stage, including 2-cell embryos, 4-cell embryos, morula, early blastocyst, blastocyst, expanded blastocyst and hatched blastocyst.

As a control group, female mice from the C57Bl/6 lineage were used. They underwent the same procedures as the other groups except for vitrification since their embryos were freshly cultured. The number of embryos strains and respective control were B6.129SVEV-CCBP2-D6 (n=31/74), B6.129P2-Nos2 (n=78/155), B6.129S2-Cd28 (n=95/79), B6.129P2-Ccl3 (n=215/95), B6.129S2-Alox5 (n=129/152), B6.129P2-Ccr2 (n=75/37), B6.129P2-Ccr5 (n=75/37) and B6.129S1-Tlr6 (n=55/85).

### Statistical analysis

Vitrification success of the transgenic lineage was evaluated in comparison to the control group. Data were analyzed using Analysis of Variance (ANOVA) followed by Tukey's test, with statistical significance set at p < 0.05.

### Ethical considerations

All procedures were conducted in compliance with the ethical principles of animal experimentation established by the Colégio Brasileiro de Experimentação Animal (COBEA) and approved by CEUA/FIOCRUZ (license number LW-33/14).

## Results

### Embryonic development of different strains

In this experiment, the development of embryos from different genetically modified strains was assessed. It is crucial to identify embryos capable of reaching the viable blastocyst stage, as this stage is crucial for implantation, organogenesis, and fetal development (Camargo et al.*,* 2017).

The embryonic development rates for the strains were as follows: D6 (55%), Nos2 (24.7%), Cd28 (45.8%), Ccl3 (50%), Alox5 (4.8%), Ccr2 (66.7%), Ccr5 (63.04%), and Tlr6 (52.8%) (Table 1).

**Table 1:** Embryonic development rates of genetically modified mouse strains after vitrification and in vitro culture.

	**2 cell embryos**	**morula**	**Bi blastocyst**	**Bl blastocyst**	**Bx blastocyst**	**Be blastocyst**	**Rate**
Nos-2	73	3	0	2	0	0	24.7%
Cont.	83	15	17	32	0	8	87.9%
D6	20	6	3	2	0	0	55%
Cont.	39	6	11	16	0	2	89.7%
Cd28	83	7	1	4	0	0	45.8%
Cont.	64	9	0	6	0	0	78.1%
Ccl3	104	18	0	14	0	0	50%
Cont.	78	7	0	10	0	0	66.67%
Tlr6	36	1	0	4	14	0	52.8%
Cont.	43	0	0	0	39	3	97.7%
Ccr2	45	13	0	17	0	0	66.7%
Cont.	20	6	0	11	0	0	85%
Ccr5	46	27	0	2	0	0	63.04%
Cont.	20	6	0	11	0	0	85%
Alox5	125	2	2	0	0	0	4.8%
Cont.	83	14	3	10	39	3	89.15%

Initial Bi- Blastocyst; Bl- Blastocyst; Bx- Expanded Blastocyst; Be- Hatched Blastocyst; Cont.- Control.

In the statistical analysis, the following genetically modified strains showed significant differences in embryonic development after embryo thawing compared to their respective control groups: Nos2 (p=0.0434), Cd28 (p=0.034), Ccl3 (p=0.0006) and Alox5 (p=0.0166). This indicates that the vitrification process and in vitro cultivation had a significant impact on the development of embryos from these strains.

On the other hand, the strains Ccr2 (p=0.0889), Ccr5 (p=0.0806), D6 (p=0,0685) and Tlr6 (p=0.0701) did not show significant differences in embryonic development after embryo thawing compared to their control groups. This suggests that these strains responded well to the vitrification process and subsequent in vitro cultivation.

## Discussion

The results of this study highlight the importance of strain-specific responses to vitrification, emphasizing the need for optimized protocols tailored to genetically modified mouse strains. The observed differences in embryonic development rates among the strains (e.g., Ccr2, Ccr5, D6, and Tlr6 showing higher resilience compared to Nos2, Cd28, Ccl3, and Alox5) align with previous findings that genotype significantly influences cryopreservation outcomes (Dinnyés et al., 1995; Byers et al., 2006). These variations may be attributed to differences in cellular metabolism, membrane composition, and mitochondrial function, which are critical factors in cryopreservation success (Amorim et al., 2011a; van Blerkom, 2011).

The superior performance of strains such as Ccr2 and Ccr5 in vitrification and subsequent embryonic development suggests inherent genetic or physiological traits that confer resistance to cryoinjury. This is consistent with studies demonstrating that certain strains exhibit greater tolerance to osmotic stress and cryoprotectant toxicity (Mullen and Critser, 2007; Tsang and Chow, 2009). Conversely, the poor performance of strains like Alox5 and Nos2 may reflect underlying vulnerabilities, such as reduced mitochondrial efficiency or increased susceptibility to oxidative stress (Eichenlaub-Ritter et al., 2011; Ramalho-Santos et al., 2009).

Creating an appropriate environment for embryonic development is essential to simulate in vivo conditions in an in vitro setting. These helps minimize the impact of factors such as media composition, pH, temperature, CO_2_ concentration, suitable culture containers, and others on the evaluation of results (Gardner and Lane, 2013).

Vitrification involves the use of a highly viscous solution and an ultrafast freezing curve, transforming cells into a glassy state. However, the high concentration of cryoprotectant agents and the duration of cell exposure to these agents can lead to osmotic stress and cell damage (Zhang, 2009). It is believed that certain strains may exhibit more resistance to this process, as observed in this study through the observed differences.

On the other hand, some studies report that the quality of oocytes at the beginning of the process is the key point to determine the potential for blastocyst formation (Rizos et al., 2002; Lonergan and Fair, 2016).

Therefore, it can be stated that the origin and environment in which oocyte growth and maturation occurs have profound impacts on the competence for embryo development (Lequarre et al., 2005). The main components of the maternal contribution to pre-implantation embryonic development are maternal transcripts and mitochondria (Moussa et al., 2015).

Mitochondrial function plays a pivotal role in embryonic development, particularly during the transition from the 2-cell stage to blastocyst formation (van Blerkom, 2011). The observed differences in development rates among strains may be linked to variations in mitochondrial activity and ATP production, which are critical for cellular homeostasis and stress response during cryopreservation (Brevini et al., 2005; Moussa et al., 2015). Strains with higher developmental competence, such as Ccr2 and Ccr5, may possess more robust mitochondrial networks capable of sustaining energy production under stress conditions (Lee and Wei, 2007; Nagai et al., 2006).

In addition to energy production, mitochondria participate in calcium signaling and apoptosis, being considered crucial for the development of embryos (Ramalho-Santos et al., 2009; Eichenlaub-Ritter et al., 2011; van Blerkom, 2011).

In these cases, failure in embryonic development may occur due to reduced ATP production (Brevini et al., 2005), pre-existing defects or accumulation of mutations in mtDNA (Keefe et al., 1995), failure to regulate oxidative stress, leading to the accumulation of reactive oxygen species (ROS) (Lee and Wei, 2007), and abnormal distribution in the cytoplasm (Nagai et al., 2006), that may be different the strain studied.

## Conclusion

This study evaluated the embryonic development and vitrification success of various transgenic mouse strains. The results demonstrated that certain transgenic strains showed significant differences in embryonic development after vitrification compared to their controls. The transgenic strains B6.129P2-Ccr2, B6.129P2-Ccr5, B6.129SVEV-CCBP2 (D6) and B6.129S1-Tlr6 exhibited the best in vitro results, indicating their higher resistance to the vitrification process.

On the other hand, the transgenic strains, B6.129P2-Nos2, B6.129S2-Cd28, B6.129P2-Ccl3 and B6.129S2-Alox 5 did not show favorable results, suggesting their lower tolerance to vitrification and in vitro cultivation.

The findings of this study emphasize the importance of selecting appropriate transgenic strains for cryopreservation and in vitro techniques, as certain strains may have inherent characteristics that affect their response to these procedures. Understanding the specific genetic factors that influence the success of vitrification and embryonic development is crucial for improving assisted reproductive technologies and preserving genetically modified animals for biomedical research.

The authors acknowledge the support and funding provided by the management of ICTB/Fiocruz for conducting the experiment and publishing the article. These contributions have facilitated advancements in the field of reproductive biotechnology and the development of more effective cryopreservation protocols.

## Data Availability

Research data is available in the body of the article.
